# Incidence of ischemic cardiovascular events in the maraviroc clinical development program

**DOI:** 10.1186/1758-2652-13-S4-P65

**Published:** 2010-11-08

**Authors:** S Portsmouth, R Burnside, A Lazzarin, M Johnson, H Valdez

**Affiliations:** 1 Pfizer Inc, New York, NY, USA; 2 Pfizer Inc, New London, CT, USA; 3 San Raffaele Scientific Institute, Milan, Italy; 4 Royal Free Hospital NHS Trust, London, UK

## Purpose of the study

Maraviroc (MVC), a CCR5 antagonist, does not appear to adversely affect serum lipids and may decrease LDL cholesterol in dyslipidemic subjects. The CCR5-del32 polymorphism, resulting in reduced (heterozygous) or absent (homozygous) CCR5 expression, is associated with reduced risk of cardiovascular disease. An early imbalance of cardiovascular adverse events was noted in the MVC phase 2b development program at 24 weeks. We investigated the longer term risk of cardiovascular events in MVC recipients in the development program. The D:A:D study and other cohorts have reported an incidence of 3.6/1000 PY for myocardial infarction (MI) among patients on HAART.

## Methods

Ischemic cardiovascular adverse events among patients treated in MERIT (MVC versus efavirenz [EFV] in treatment-naïve [TN] patients), MOTIVATE (MVC versus placebo [PBO] in treatment-experienced [TE] patients), and study 1029 (MVC versus PBO in non-R5, TE patients) were prospectively collected. Incidence rates and exposure-adjusted incidence rates (/100PY) were compared to those of comparator regimens or existing cohort data.

## Summary of results

**Figure 1:**
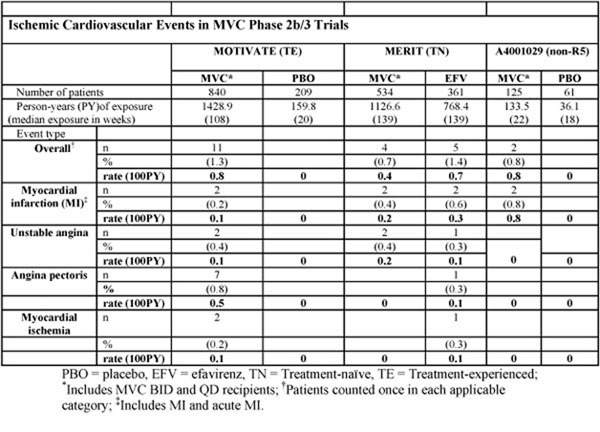


## Conclusions

Rates of ischemic cardiovascular events were similar in TN patients treated with either MVC or EFV. In TE patients, the rate in the MVC arm was low and there were no events in the PBO arm. However, exposure to maraviroc was much greater than PBO in the TE population, and most events occurred in individuals with known cardiovascular risk factors. There were no additional patients with MI beyond the first 24 weeks among TE patients. Rates of MI in the TE population (1.0/1000PY) were consistent with published rates from prospective cohort studies.

